# Cerebral Cavernous Malformations: Review of the Genetic and Protein–Protein Interactions Resulting in Disease Pathogenesis

**DOI:** 10.3389/fsurg.2016.00060

**Published:** 2016-11-14

**Authors:** Jacob F. Baranoski, M. Yashar S. Kalani, Colin J. Przybylowski, Joseph M. Zabramski

**Affiliations:** ^1^Department of Neurosurgery, St. Joseph’s Hospital and Medical Center, Barrow Neurological Institute, Phoenix, AZ, USA

**Keywords:** cavernous malformation, CCM, *CCM1*, *CCM2*, *CCM3*, *KRIT1*, *PDCD10*

## Abstract

Mutations in the genes *KRIT1, CCM2*, and *PDCD10* are known to result in the formation of cerebral cavernous malformations (CCMs). CCMs are intracranial lesions composed of aberrantly enlarged “cavernous” endothelial channels that can result in cerebral hemorrhage, seizures, and neurologic deficits. Although these genes have been known to be associated with CCMs since the 1990s, numerous discoveries have been made that better elucidate how they and their subsequent protein products are involved in CCM pathogenesis. Since our last review of the molecular genetics of CCM pathogenesis in 2012, breakthroughs include a more thorough understanding of the protein structures of the gene products, involvement with integrin proteins, and MEKK3 signaling pathways, and the importance of CCM2–PDCD10 interactions. In this review, we highlight the advances that further our understanding of the “gene to protein to disease” relationships of CCMs.

## Introduction

Cerebral cavernous malformations (CCMs) are intracranial lesions comprised of low flow and abnormally dilated capillary endothelial channels with increased permeability that predispose these vessels to episodes of thrombosis and focal hemorrhage, resulting in seizures and neurologic deficits.

Loss-of-function mutations in the genes Krev interaction trapped 1 (*KRIT1* or *CCM1*), cerebral cavernous malformation 2 (*CCM2*), and programed cell death protein 10 (*PDCD10* or *CCM3*) result in the formation of CCMs. Although a role for these three genes in the formation of these intracranial vascular lesions has been established since the 1990s, additional works have further elucidated the molecular mechanisms by which mutations in these genes and the resultant aberrant proteins interact, leading to the formation of CCMs.

The three CCM proteins coded by *KRIT1, CCM2*, and *PDCD10* form a trimeric protein complex. Germline loss-of-function mutations in any of these genes may lead to the formation of CCMs. Therefore, it is reasonable to assume that a molecular pathway exists that requires all three proteins to function together correctly for proper cellular function. Moreover, research is demonstrating how each component protein is capable of interacting with numerous other signaling and cytoskeletal molecules allowing for a diverse range of functions in molecular signaling pathways *via* unique protein–protein interactions.

In this review, we highlight some of these recent advances that further our understanding of the “gene to protein to disease” relationships of CCMs. This work is meant to expand upon and to serve as an update to the previous review from this institution published in 2012 (1).

### KRIT1 (CCM1)

In 1994, Kurth et al. were the first to begin successful mapping of a causative gene for CCMs (2). Utilizing linkage analysis, these authors identified the q11–q12 region of chromosome 7, specifically a 33-centimorgan (cM) region from D7S502 to D7S479, as potentially being responsible for CCM formation in a large Hispanic family. Concurrently, Marchuk et al. identified linkage between CCM and a sequence on the proximal long arm of chromosome 7 between D7S502 and D7S515 (3), and Gunel et al. successfully identified the D7S699 locus as linked to CCM (4). The potential region for the precise location of the *CCM1* gene was further refined to a 4-cM segment of the human 7q21-q22, bounded by D7S2410 and D7S689 (5, 6).

Krev interaction trapped 1 was subsequently identified as the *CCM1* gene in an analysis of multiple affected Hispanic families (7, 8). This finding was confirmed in a 1999 study involving French families with hereditary CCMs (9).

Krev interaction trapped 1 encodes the 736-amino acid peptide. It is the largest of the three CCM proteins and is comprised of an N-terminal Nudix domain with three NPxY/F motifs, an ankyrin repeat region, and a C-terminal FERM domain (band 4.1 protein, ezrin, radixin, and moesin) (10). Although KRIT1 has no known catalytic activity, it binds and interacts with scaffolding and signaling molecules.

### KRIT1 and Integrin Activation

Krev interaction trapped 1 interacts with integrin cytoplasmic-associated protein-1 (ICAP1) (10). Integrins are transmembrane receptors whose functions include cellular attachment to the extracellular matrix as well as established roles in cell-to-cell signal transduction, embryogenesis, and tissue formation and repair. ICAP1 is one of the few established suppressors of integrin activation (10, 11). The N-terminal of KRIT1 binds to ICAP1 *via* the first of its three NPxY/F motifs and an unpredicted binding motif encompassing residues H172 to R185 (10). Liu et al. demonstrated that ICAP1 utilizes the same binding domain to interact with KRIT1 as it does with β-integrin peptides (10). Therefore, ICAP1 cannot bind to integrin and suppress its activation when it is bound to KRIT1, resulting in increased integrin activation while ICAP1 is bound to KRIT1.

Interestingly, it also appears that KRIT1 stabilizes ICAP1 and that the loss of KRIT1 results in decreased ICAP1 levels and, therefore, increased integrin activation (12). Faurobert et al. found that the loss of KRIT1 or CCM2 resulted in ICAP1 destabilization and a subsequent increase in β1 integrin activity (12). Furthermore, they found that endothelial cells that are lacking KRIT1 or CCM2 do not properly interact with the extracellular matrix, and that this anomalous interaction with the extracellular matrix may impair endothelial barrier function and result in increased RhoA-dependent contractility.

It is possible that mutations leading to aberrant KRIT1–ICAP1 interactions could produce abnormal integrin activation and consequently disrupt normal tissue development. Although the precise role of the KRIT1–ICAP1 interaction on integrin activity in endothelial cells has not been fully elucidated, it is clearly an area that warrants further investigation.

Work by Renz et al. has demonstrated that CCM proteins are involved in the modulation of the β1-integrin signaling cascade that regulates angiogenesis (13). These authors showed that loss of CCM proteins in endothelial cells results in the β1-integrin-dependent overexpression of the transcription factor, Krüppel-like factor-2 (KLF2). This overexpression of KLF2 subsequently results in increased activation of epidermal growth factor-like domain-containing protein 7 (EGFL7) and angiogenesis. This work suggests that CCM proteins are critical regulators of endothelial quiescence, and that loss of proper CCM signaling can result in aberrant angiogenesis. It is possible that this CCM-mediated regulation of KLF2 is further regulated by a CCM2–MEKK3 [mitogen-activated protein kinase kinase kinase-3 (MAP3K3)] interaction (14–16).

Krev interaction trapped 1 localizes at endothelial cell–cell junctions. The loss of KRIT1 results in impaired endothelial cell–cell junctions and loss of integrity associated with increased Ras homolog gene family (member A), RhoA, and protein activity (17–20).

### KRIT1 and the Heart of Glass Receptor

The heart of glass receptor (HEG) is a transmembrane protein that plays a role in cardiovascular development. The loss of HEG results in aberrant cardiovascular morphogenesis (19, 21). Work by Gingras et al. demonstrated that the C-terminal FERM domain of KRIT1 binds to HEG and that this interaction is critical for the proper localization of KRIT1 at endothelial cell–cell junctions (19). Inhibition of this interaction results in failure of KRIT1 to localize at the endothelial cell–cell junctions, which results in aberrant cardiovascular development.

Interestingly, recent work by Zheng et al. demonstrated that postnatal mice with conditional knockout of HEG in endothelial cells do not form cavernous malformations (21). However, postnatal mice with conditional knockout of CCM2 in endothelial cells rapidly develop CCMs in the central nervous system. These authors were also able to demonstrate the absence of HEG mutations in a cohort of human patients with sporadic CCMs (sporadic CCMs are single isolated lesions that occur in the absence of germline mutations in *KRIT1, CCM2*, or *PDCD10*). Together, these findings suggest that HEG–CCM interactions are critical for embryonic cardiovascular development and growth and that CCMs arise due to postnatal HEG-independent CCM signaling aberrations in the endothelium of the central nervous system.

### KRIT1 and Notch Signaling

Krev interaction trapped 1 is also linked to Notch signaling. Wustehube et al. found that KRIT1 inhibition results in decreased Notch pathway activity while KRIT1 overexpression leads to upregulation of the Notch pathway as demonstrated by increased DLL4 expression (22). This reduction in Notch signaling in KRIT1-deficient endothelial cells results in irregular vascular sprouting and abnormal angiogenesis. Schulz et al. demonstrated that silencing of KRIT1 in endothelial cells resulted in decreased Notch3 activity in cocultured brain pericytes (23). Additionally, these authors found that DLL4 proteins stimulated Notch3 receptors on human brain pericytes and that activated Notch3 induced the expression of *PDGFRB2, N-Cadherin, HBEGF, TGFB1, NG2*, and *S1P* genes. Upregulated Notch3 signaling in pericytes promoted proper pericyte–endothelial cell interactions, stimulating proper angiogenesis (23). Pericytes devoid of functional Notch3 signaling failed to suppress aberrant angiogenesis adequately. Therefore, proper Notch–KRIT1 interactions and subsequent endothelial cell–pericyte interactions are important to maintain proper vascular development, and dysregulation of Notch signaling and the Notch3–KRIT1 interaction may contribute to the pathogenesis of CCMs.

### KRIT1 and Regulation of Reactive Oxygen Species

Krev interaction trapped 1 also interacts with pathways that regulate the degradation of reactive oxygen species. A lack of KRIT1 results in the decreased expression of superoxide dismutase-2, a reactive oxygen species scavenging molecule, which leads to increased levels of reactive oxygen species, AKT (protein kinase B) phosphorylation, and AKT-dependent forkhead box protein O1 (FOXO1) phosphorylation (24).

Choquet et al. have demonstrated that increased levels of reactive oxygen species and oxidative stress, as marked by deregulation of cytochrome P450, may contribute to increased severity of CCM disease (25). These authors found that patients with concomitant mutations in the cytochrome P450 family of proteins and CCMs tended to have more lesions, larger lesions, and higher rates of intracranial hemorrhage.

### CCM2 and PDCD10

The identification of families with hereditary CCMs, but no *KRIT1* mutations, highlighted the possibility of the involvement of genetic loci other than *KRIT1* in the pathogenesis of CCMs (26, 27). Indeed, evidence began to emerge that linked two additional chromosomal regions in families with CCMs – one segment on 7p and one on 3q (26). The *CCM2* gene was successfully mapped to 7p15-p13, spanning an 11-cM region between D7S2846 and D7S1818. Within in this region, Liquori et al. identified eight genes that were the most likely to be involved in CCM pathogenesis, including one gene that contains a phosphotyrosine-binding domain and was predicted to interact with *KRIT1* (28). In 2004, the *CCM2* gene was confirmed by Denier et al. and was identified as being located on 7p13, containing 10 coding exons (27). *CCM2* codes for the 444-amino acid protein CCM2/malcavernin, which contains a predicted N-terminus phosphotyrosine-binding domain and a C-terminal helical domain (28, 29). Endothelial cells require CCM2 for proper cytoskeletal structure, cell–cell interactions, and vessel lumen formation *via* the interaction of many critical signaling pathways.

The third CCM locus, PDCD10/CCM3, is located on 3q25.2-27. This locus was identified within a 22-cM interval flanked by D3S1763 and D3S1262 (26). The role of *PDCD10* in CCM pathogenesis was first proposed in 2005 by Bergametti et al. (30). They screened 20 unrelated families with CCMs, but found no mutations in *KRIT1* or *CCM2*.

Programed cell death protein 10, located on 3q26.1, is a highly conserved gene containing seven coding and three non-coding exons, which result in a 212-amino acid protein (PDCD10). This protein is ubiquitously expressed and has an N-terminal dimerization domain and a C-terminal focal adhesion targeting (FAT)-homology domain (31). PDCD10 is the third member of the CCM protein complex and binds directly to CCM2 as well as several other signaling molecules (31, 32).

### CCM2, CCM2L, and Interactions with the MEKK3 Pathway

One of the roles of CCM2 in the CCM signaling pathway is to solidify endothelial cell–cell junctions and stabilize vascular structures. A significant development in the study of CCM2 and its functions was the discovery of its paralog. Termed CCM2-like (CCM2L), this peptide has a high sequence homology to CCM2 and is selectively expressed in endothelial cells during periods of angiogenesis (33). Although there appears to be some similarity in their functions, the loss of CCM2L in *Xenopus* (frog) results in a phenotype similar to that of CCM2 knockouts, and the CCM2L-null phenotype can be rescued by overexpression of CCM2 (34); the two molecules are not entirely homologous. Because the three CCM proteins bind to each other to form a trimeric complex, CCM2L directly competes with CCM2 for binding to KRIT1, and therefore, subsequently inhibits CCM2-mediated endothelial cell–cell adhesion stability by uncoupling these upstream components of the CCM pathway from CCM3 (33). Interestingly, CCM2L does not compete with CCM2 for binding to PDCD10. These results suggest that CCM2L and subsequent modulation of the CCM pathway are molecular mechanisms by which endothelial cells maintain vessel stability and induce postnatal vessel growth.

Work by Cullere et al. in 2015 further elucidated the relationship between CCM2 and CCM2L and their mechanisms of action (15). These authors demonstrated that both CCM2 and CCM2L could bind to and inhibit MEKK3 in a complex with KRIT1. MEKK3 and its downstream targets and effectors function in key signaling pathways, including those involved in endothelial–mesenchymal transition, cell proliferation, and cellular migration. Moreover, the MEKK3 pathway plays a critical role in early cardiovascular development (35). Binding of both CCM2 and CCM2L to MEKK3 inhibits its activation and prevents its ability to phosphorylate MEK5 (dual specificity mitogen-activated protein kinase kinase 5), a downstream target. Lack of CCM2 also results in increased activation of extracellular regulated kinase 5 (ERK5), a mitogen-activated protein kinase 5, in endothelial cells. ERK5 is ubiquitously expressed in endothelial cells, where it is thought to play a role in cell survival and maturation. These findings suggest that both CCM2 and CCM2L are capable of regulating the activity of MEKK3, and therefore augmenting multiple major signaling pathways that have essential cellular functions.

The mechanism of CCM2 interaction with MEKK3 was discovered in 2015 by Wang et al. (16). They described the crystal structure of the C-terminus of the CCM2 peptide and found that it contains a five-helix domain followed by a C-terminal tail that forms a separate, isolated helix capable of interacting with the other five helical domains. Furthermore, they discovered that the MEKK3 N-terminal helix binds the C-terminus of the CCM2 peptide, successfully revealing the mechanism for CCM2-MEKK3 interaction and signaling.

Zhou et al. determined the role of CCM proteins in the MEKK3 pathway (14). They demonstrated that CCM deficiency results in increased endothelial cell expression of transcription factors KLF2 and KLF4 *via* lack of inhibition of the MEKK3 signaling cascade. They also showed that exogenous inhibition of MEKK3 in CCM-deficient organisms is capable of rescuing the CCM-deficient phenotype.

### CCM2–PDCD10 Binding and Mutual Complex Stabilization

Perhaps the most intriguing recent discovery involving the CCM proteins includes the interaction between CCM2 and PDCD10. The three CCM proteins bind together to form a trimeric molecule, and while the binding relationships between KRIT1 and CCM2 – and its paralog CCM2L – have been well described (33, 34), the molecular mechanism and functional importance of the CCM2-PDCD10 binding were only recently identified. Draheim et al. successfully demonstrated both the mechanism of interaction between the CCM2 and PDCD10 molecules and that this interaction is required for their activity (36).

The crystalline structure of PDCD10 has an N-terminal dimerization domain and a C-terminal FAT-homology domain. Within this FAT domain is a highly conserved surface termed “hydrophobic patch 1” (HP1) that is critical for PDCD10 binding with numerous molecules including CCM2 (31, 37). However, the mechanism by which this domain interacted with CCM2 was unknown. Draheim et al. showed that the structure of CCM2 contains short helical sequences called LD motifs, and it is at these LD motifs that CCM2 binds PDCD10 *via* its highly conserved HP1 region of the PDCD10 FAT domain (36). Furthermore, the authors demonstrated that this binding stabilizes the CCM2–PDCD10 complex and prevents proteasomal degradation of the protein complex. The loss of proper binding of CCM2 to PDCD10 due to aberrations in the binding domains results in abnormal endothelial cell function, demonstrating that these domains are both essential for CCM2–PDCD10 interaction, CCM protein signaling, and endothelial cell function (36).

## Additional Developments

### KRIT1, CCM2, PDCD10, and RhoA–ROCK Signaling

Loss of KRIT1 function results in impairment of endothelial cell–cell junctions, with a loss of integrity and an associated increase in RhoA activity (17–20). Activated RhoA levels are also increased in endothelial cells lacking the normal function of CCM2 or PDCD10 (17, 38–40).

Activated RhoA results in actin polymerization and stress fiber formation, in part *via* the RhoA effector molecule Rho-associated coiled-coil-forming kinase (ROCK). ROCK, a serine-threonine kinase, polymerizes actin and increases actomyosin contractility *via* inhibition of myosin light chain (MLC) phosphatase. Inhibition of any of the CCM proteins results in increased levels of phosphorylated MLC, increased stress fiber formation, and the inability of endothelial cells to properly migrate, form three-dimensional tubal structures, and create a stable impermeable monolayer (20, 39–41). All of these anomalies in CCM-knockout mice were successfully rescued by inhibition of ROCK, further supporting the role of RhoA–ROCK signaling in the CCM phenotype (20, 39–41).

The precise mechanism by which CCM proteins interact with the RhoA pathway remains to be fully elucidated. Some possibilities include the interaction of KRIT1 with β1 integrin signaling (10, 12).

Cerebral cavernous malformation 2 may selectively promote E3 ubiquitin ligase-mediated degradation of RhoA *via* interaction with Smad ubiquitin regulatory factor 1 (SMURF1) (42). Crose et al. found that cells lacking CCM2 possessed increased levels of RhoA, but not increased levels of other known SMURF1 substrates, indicating that disruption of CCM2 does not inhibit SMURF1 itself, but rather the interaction of SMURF1 with RhoA (42).

Zheng et al. showed that RhoA activity increases when STK25 (a GCKIII serine-threonine kinase and known binding partner of PDCD10) is knocked down, which could be a potential mechanism for increased RhoA activation in PDCD10-deficient endothelial cells (38).

### PDCD10 and Neuronal Migration

Additional functions of CCM proteins are being identified. Louvi et al. discovered that PDCD10 has a pivotal role in neuronal migration *via* suppression of RhoA signaling. They demonstrated that PDCD10 activity is required for proper radial glia and pyramidal neuron migration through the subventricular zone (43). Loss of PDCD10 resulted in dysregulation of the actin and microtubule cytoskeleton and adversely affected cellular morphology and migration. This dysregulation may be a result of CCM-mediated regulation of RhoA signaling.

### PDCD10 Mutations Associated with Increased CCM Severity

Although loss-of-function mutations in any of the three CCM genes may result in CCM formation, different mutations result in varying degrees of disease severity. Patients with CCMs harboring PDCD10 mutations have a significantly greater disease burden and severity compared to those with KRIT1 or CCM2 mutations. Cigoli et al. found that patients with PDCD10 mutations had an earlier onset of disease symptomology compared to those with KRIT1 or CCM2 mutations. Shenkar et al. demonstrated that patients with familial PDCD10 mutations had a significantly more aggressive clinical CCM disease phenotype than patients with KRIT1 or CCM2 familial disease or sporadic lesions (44). Patients with PDCD10 mutations had an increased number of lesions and also presented with lesion hemorrhages earlier in life. Moreover, these authors found additional PDCD10 aberrations in addition to the CCMs, including scoliosis, cognitive disability, and skin lesions, further suggesting that PDCD10 plays other roles in tissue development aside from endothelial cell formation (43, 44).

### PDCD10 and Meningiomas

Programed cell death protein 10 mutations are becoming increasingly identified in other disorders of tissue development. A particularly exciting discovery is the predisposition of patients with PDCD10 mutations to develop meningiomas in addition to CCMs. Several reports in the literature demonstrate that patients with familial PDCD10 mutations have developed late–onset meningiomas in addition to multiple CCMs (45–47). Such reports highlight the potential functional diversity of CCM proteins in tissue development.

### Endothelial-to-Mesenchymal Transition in CCMs and Potential Role of Anti-inflammatory Agents

Another recent intriguing development in the study of CCMs is the discovery that PDCD10-deficient endothelial cells in CCMs undergo endothelial-to-mesenchymal transformation (48). This transformation is the result of the loss of PDCD10-mediated regulation and subsequent upregulation of β-catenin signaling. Bravi et al. also found that once this change occurred in the endothelial cells of CCMs, TGF-β/BMP signaling was subsequently required for the progression of the disease (48). The authors also found that this endothelial-to-mesenchymal cell transformation occurred in sporadic CCM lesions in addition to the familial and animal model lesions (49). While these findings are interesting from a pathogenic standpoint, they are even more intriguing because they suggest potential therapeutic options for the treatment and prevention of CCMs. Indeed, Bravi et al. found that the anti-inflammatory drugs sulindac sulfide and sulindac sulfone, which attenuate β-catenin transcription activity, reduced aberrant vascular malformations in a murine PDCD10-deficient model of CCMs (48).

## Conclusion

Significant research findings from 2000 to 2015 have further enhanced our understanding of the pathogenesis of CCM formation. The use of advanced sequencing technologies to characterize genomic mutations and the identification of new signaling pathways and protein–protein interactions have led to great strides in understanding the molecular genetics involved in the development of CCMs. However, many unanswered questions remain, and future studies are clearly needed to improve our understanding of CCM pathogenesis. “Gene to protein to disease” mechanisms involved in the pathogenesis of CCMs should shed further light on potential therapeutic targets.

## Author Contributions

All the authors made substantial contributions to the conception or design of the work.

## Conflict of Interest Statement

The authors declare that the research was conducted in the absence of any commercial or financial relationships that could be construed as a potential conflict of interest. The reviewer BC and handling Editor declared their shared affiliation, and the handling Editor states that the process nevertheless met the standards of a fair and objective review.

## Abbreviations

CCM, cerebral cavernous malformation; FAT, focal adhesion targeting; HP1, hydrophobic patch 1; ICAP1, integrin cytoplasmic-associated protein-1; MLC, myosin light chain; ROCK, Rho-associated coiled-coil-forming kinase; SMURF1, Smad ubiquitin regulatory factor 1.
