# Corrigendum: Cerebral Cavernous Malformations: Review of the Genetic and Protein–Protein Interactions Resulting in Disease Pathogenesis

**DOI:** 10.3389/fsurg.2017.00031

**Published:** 2017-07-18

**Authors:** Jacob F. Baranoski, M. Yashar S. Kalani, Colin J. Przybylowski, Joseph M. Zabramski

**Affiliations:** ^1^Department of Neurosurgery, St. Joseph’s Hospital and Medical Center, Barrow Neurological Institute, Phoenix, AZ, United States

**Keywords:** cavernous malformation, CCM, *CCM1*, *CCM2*, *CCM3*, *KRIT1*, *PDCD10*

In the original article, the reference *Cigoli et al*. is missing from the section “Additional Developments”, sub-section “*PDCD10 Mutations Associated with Increased CCM Severity*”.

The text of the subsection should read:

Although loss-of-function mutations in any of the three CCM genes may result in CCM formation, different mutations result in varying degrees of disease severity. Patients with CCMs harboring PDCD10 mutations have a significantly greater disease burden and severity compared to those with KRIT1 or CCM2 mutations. Cigoli et al. found that patients with PDCD10 mutations had an earlier onset of disease symptomology compared to those with KRIT1 or CCM2 mutations (50). Shenkar et al. demonstrated that patients with familial PDCD10 mutations had a significantly more aggressive clinical CCM disease phenotype than patients with KRIT1 or CCM2 familial disease or sporadic lesions (44). Patients with PDCD10 mutations had an increased number of lesions and also presented with lesion hemorrhages earlier in life. Moreover, in addition to the CCMs, these authors found PDCD10 aberrations, including scoliosis, cognitive disability, and skin lesions, further suggesting that PDCD10 plays other roles in tissue development aside from endothelial cell formation (43, 44).

The authors apologize for this error and state that this oversight does not change the scientific conclusions of the article in any way.

## Conflict of Interest Statement

The authors declare that the research was conducted in the absence of any commercial or financial relationships that could be construed as a potential conflict of interest.
